# Analysis of simultaneous modulated accelerated radiotherapy (SMART) for nasopharyngeal carcinomas

**DOI:** 10.1093/jrr/rru012

**Published:** 2014-03-09

**Authors:** Jian Min Tang, Xiu Mei Ma, Yan Li Hou, Li Yan Dai, Hong Bin Cao, Ming Ye, Yong Rui Bai

**Affiliations:** Department of Radiation Oncology, Renji Hospital, School of Medicine, Shanghai Jiao Tong University, 200127, Shanghai, China

**Keywords:** intensity-modulated radiotherapy, nasopharygeal carcinoma, prognosis, simultaneous modulated accelerated radiotherapy, toxicity

## Abstract

The purpose of this study was to analyze the clinical outcomes of simultaneous modulated accelerated radiotherapy (SMART) in patients with nasopharyngeal carcinoma (NPC). A total of 97 patients who underwent SMART for NPC between August 2005 and November 2011 were evaluated. The prescribed dose was 69.9 Gy/30 fractions at 2.33 Gy/fraction to the primary gross tumor volume (PGTV) including the nasopharynx gross target volume and the positive neck lymph nodes, and 60 Gy/30 fraction at 2.0 Gy/fraction to the PCTV1; 54 Gy/30 fractions at 1.8 Gy/fraction was given to the PCTV2. Among 59 patients with local advanced disease, 31 patients received concurrent chemoradiotherapy (chemo-RT) with a regimen consisting of 135 mg/m^2^ paclitaxel on Day 1 and 25 mg/m^2^ cisplatin on Days 1–3. The median follow-up period was 42 months. The local control rate (LCR), distant metastases-free survival (DMFS) and overall survival (OS) rates were 93.3%, 90.3% and 91.6% at 3 years, and 87.6%, 87.9% and 85.7% at 5 years, respectively. There was no significant difference in outcome with respect to these three indicators for Stage III and IV disease treated with/without concurrent chemoradiotherapy (*P* > 0.05). Acute toxicities included Grade 3 mucositis, skin desquamation, and leucopenia, which occurred in 78 (80.4%), 8 (8.2%), and 45 (46.4%) patients, respectively. No patient had a Grade 3–4 late toxicity. SMART was associated with a favorable outcome for NPC with acceptable toxicity. The local-regional control was excellent but distant metastasis remains the main risk. The combination of SMART and chemotherapy needs to be optimized through further studies to enhance outcomes for locally advanced diseases.

## INTRODUCTION

Radiotherapy is the most effective treatment for nasopharyngeal cancer (NPC). However, local recurrence and distant metastasis are not uncommon, especially for locally advanced cases. Historically, the local control rate (LCR) for locally advanced disease ranges from 44–68% [1–4]. As radiotherapy has progressed significantly over the past decade, with combined modality therapy the LCR has increased to 75–95% for locally advanced disease [5–9].

We sought to improve the cure rate by increasing the biologically effective dose to the boost volume, and designed a regimen of 2.33 Gy for a total of 69.9 Gy with the use of IMRT with SMART boost techniques. The aim of our study was to evaluate the long-term local control, survival, and concomitant toxicities of this technique delivered at our dose scheme, with or with concurrent chemotherapy, for the treatment of NPC.

## MATERIALS AND METHODS

### Common conditions

Between August 2005 and November 2011, a total of 97 eligible patients were enrolled in this study. The patients had to fulfill all of the following criteria including: (i) histologically confirmed NPC by biopsy, (ii) no evidence of distant metastasis, (iii) no previous treatment for NPC, (iv) adequate liver and renal function, (v) adequate bone marrow function, and (vi) Karnofsky performance status ≥ 80.

The pretreatment evaluation consisted of a complete history and physical examination together with a nasopharyngoscopy, chest radiography, ultrasonography of the abdominal region, and hematologic and biochemical profiles. Additional investigations including emission computed tomography (ECT) and positron emission tomography (PET) were performed before initiation of treatment if there was a suspicion of metastasis. After March 2009, magnetic resonance imaging (MRI) of the nasopharynx was required to determine stage unless there was a contraindication. Prior to that time, MRI and contrast-enhanced computed tomography (CT) of the nasopharynx were alternatively performed to determine stage. As this trial was initiated in September 2005, patients were initially staged according to the 2002 AJCC/UICC system in our protocol. After the publication of the 2009 AJCC/UICC system, all of the enrolled patients were restaged according to the 2009 AJCC/UICC criteria. The demographic and clinical characteristics of the patients are shown in Table 1. Written informed consent was obtained from all patients. The protocol was approved by the ethics committees of the participating institutions, and the trial was conducted in accordance with the Declaration of Helsinki.

**Table 1.** Patient and disease characteristics of 97 patients

Characteristic	*n*	%
Gender		
Male	65	67.00%
Female	32	33.00%
Age (years)^a^	50 (8–79)	
Histology		
WHO I	4	4.10%
WHO II	11	11.40%
WHO III	82	84.50%
Tumor classification		
By CT	26	26.80%
By MRI	53	54.60%
By CT + MRI + PET	18	18.60%
T classification^#^		
T1	24	24.70%
T2	35	36.10%
T3	17	17.50%
T4	21	21.70%
N classification^b^		
N0	27	27.80%
N1	33	34.00%
N2	29	29.90%
N3	8	8.30%
Clinical stage^b^		
I	9	9.30%
II	29	29.90%
III	34	35.00%
IVa, IVb	25	25.80%

WHO = World Health Organization, CT = computed tomography, MRI = magnetic resonance imaging, PET = positron emission tomography. ^a^Median (range). ^b^Determined according to the 7th American Joint Committee on Cancer and the International Union Against Cancer staging system.

### Treatment

Patients were immobilized in the supine position with a thermoplastic mask from head to neck, and a contrast-enhanced CT simulation was performed with a slice thickness of 3 mm extending from the vertex to carina of the trachea. If possible, fusion of MRI and/or PET with the planning CT images was performed to aid in the delineation of the gross tumor volume (GTV). All target volumes were outlined slice by slice on the axial contrast-enhanced CT images in the treatment planning system. The target volumes were defined in accordance with the International Commission on Radiation Units and Measurements Reports 50 and 62 [10–11]. The GTV included the primary disease and nodes greater than 1 cm in diameter or nodes with necrotic centers. The clinical target volume (CTV) was divided into two parts: CTV1 and CTV2. CTV1 was defined as the nasopharynx gross target volume plus a 5–8-mm margin (1–3-mm margin posteriorly) to encompass the high-risk sites of microscopic extension and the whole nasopharynx. CTV2 was defined as the CTV1 plus a 5–10-mm margin (1–3-mm margin posteriorly) to encompass the low-risk sites of microscopic extension, the level of the lymph node located, and the elective neck area (bilateral retropharyngeal and Levels Ib, II, III and Va are routinely covered for all N0 patients, whereas bilateral Levels IV and Vb were also included for N1 patients). Planning target volumes (PTVs) for all GTVs and CTVs were generated automatically with a 3–5-mm margin after delineation of tumor targets in order to include biological and technical uncertainties. The margin was applied in all dimensions except posteriorly, where a 1–3-mm margin was used because there is less biological uncertainty at the skull.

A total of 97 patients who underwent SMART for NPC between August 2005 and November 2011 were evaluated. The prescribed dose was 69.9 Gy/30 fractions at 2.33 Gy/fraction to the PGTV (including the nasopharynx gross target volume and the positive neck lymph nodes) and 60 Gy/30 fractions at 2.0 Gy/fraction to the PCTV1; 54 Gy/30 fractions at 1.8 Gy/fraction was given to the PCTV2. In our department, we use the step and shoot technique for NPC patients, with normally nine beams to ensure that the dose distribution in the target region is quite uniform. The description regarding dose specification in our treatment planning of intensity-modulated radiotherapy (IMRT) should meet the following conditions: the >95% of the PGTV should receive the prescribed dose; ≤10% of the PGTV should receive >110% of the prescribed dose; 100% of the GTV should receive the prescribed dose; > 95% of the PCTV1 should receive the prescribed dose; > 95% of the PCTV2 should receive the prescribed dose. All of the critical adjacent structures, including the brainstem, spinal cord, temporal lobe, cochlea, lens, optic nerves, optic chiasm, pituitary gland, parotid glands, oral cavity, larynx and temporo-mandibular joints (TMJs), were carefully outlined in three dimensions. The dosage to these structures was reduced as much as possible, without sacrificing coverage of the tumor target. The ability to spare these structures depended on the extent and location of the tumor. The maximum dosage to these structures was restricted as follows so as not to exceed their tolerance doses: 54 Gy for the brainstem; 45 Gy for the spinal cord; 60 Gy for the temporal lobes; 5 Gy for the lens; 60 Gy for the cochlea; 54 Gy for the optic nerves and chiasm; 50 Gy for the larynx and TMJs; and a mean dose of < 30 Gy for the parotids.

For patients with extensive locoregional disease, the addition of concurrent chemotherapy is a standard recommendation based on the survival benefit [12]. In our study, 59 patients with extensive locoregional disease (Stage III, IVa and IVb) were divided into two groups at random: one group receiving concurrent chemotherapy and the other group receiving induction chemotherapy only and/or adjuvant chemotherapy. The regimen consisted of 135 mg/m^2^ paclitaxel on Day 1 and 25 mg/m^2^ cisplatin on Days 1–3 instead of cisplatin for concurrent chemotherapy, and the same regimen was also applied for induction chemotherapy and adjuvant chemotherapy. Induction chemotherapy was given in one to two cycles and adjuvant chemotherapy given in four to six cycles. Patients in the concurrent chemotherapy group received one cycle of chemotherapy which started simultaneously with radiotherapy, and whenever possible these patients also received induction chemotherapy and/or adjuvant chemotherapy. Comparisons of the demographic and clinical characteristics of the two groups are shown in Table 2.

**Table 2.** Patient characteristics of the two groups

Variables	Concurrent group (*n* = 31 patients)	No concurrent group (*n* = 28 patients)	*P*-value
*n*	31	28	
Age (years)			0.29
≤60	27	20	
>60	4	8	
Gender			0.41
Male	24	19	
Female	7	9	
Clinical stage			0.94
Stage III	18	16	
Stage IVa	13	12	

### Follow-up and statistical analysis

Planned patient assessment post-treatment included indirect nasopharyngeal speculum examinations and a fiberoptic nasopharyngoscopy every 3 months for the first year of follow-up, every 6 months in the second and third years, and annually thereafter. A follow-up CT or MRI scan of the nasopharynx and neck was performed approximately every 6 months from the completion of radiation therapy for the first 3 years. Afterward, an annual CT or MRI scan was obtained. During every follow-up visit, disease status and treatment toxicity were assessed. A suspected recurrence was histologically proven unless a biopsy would cause an excessive risk of injury to the patient. Biochemistry profiles, chest radiography and abdominal ultrasonography were routine elements of the assessment. Further investigations were arranged as indicated. Patients were classified as progression free as long as they remained alive without local, regional or distant recurrences.

The Kaplan–Meier method was used to calculate actuarial rates of local control, distant metastases-free survival (DMFS), and overall survival (OS). Late toxicities were graded according to the Radiation Therapy Oncology Group radiation morbidity scoring criteria [13]. All time-to-failure endpoints were calculated from the date of the end of treatment to the date of failure/last follow-up for patients who did not experience failure. Patients who developed distant metastases were still observed for locoregional failure.

## RESULTS

### Local control and survival

Radiation therapy was completed without interruption in all patients. Every patient received the prescription dose of 69.9 Gy to the gross disease and was regularly followed up until death or 31 December 2012. Of the total patients, 42 received radiotherapy together with concurrent chemotherapy as planned, 19 received radiotherapy with no chemotherapy, and 36 received only induction/adjuvant chemotherapy. The median duration of follow-up was 42 months (range, 13–88 months).

**Fig. 1.**
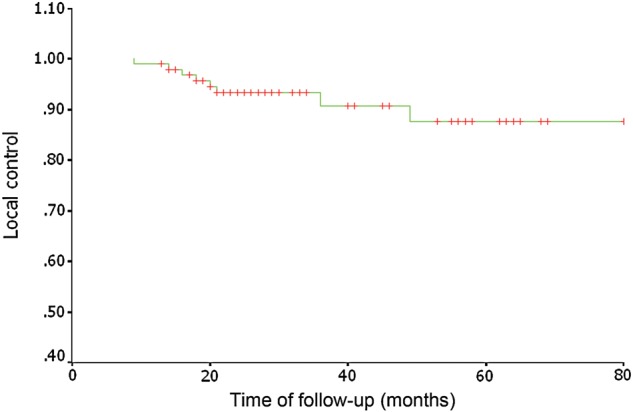
Local control for the 97 patients treated with SMART.

**Fig. 2.**
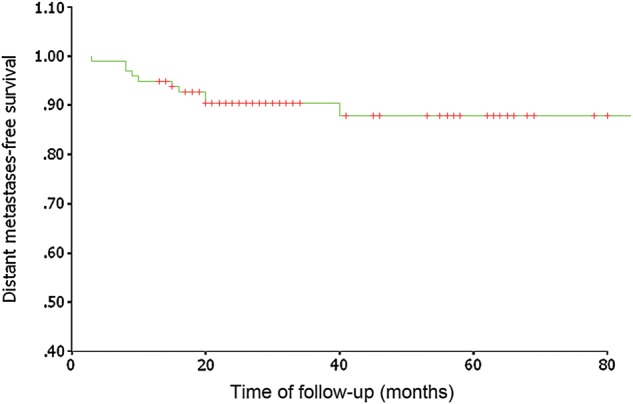
Distant metastases-free survival for the 97 patients treated with SMART.

**Fig. 3.**
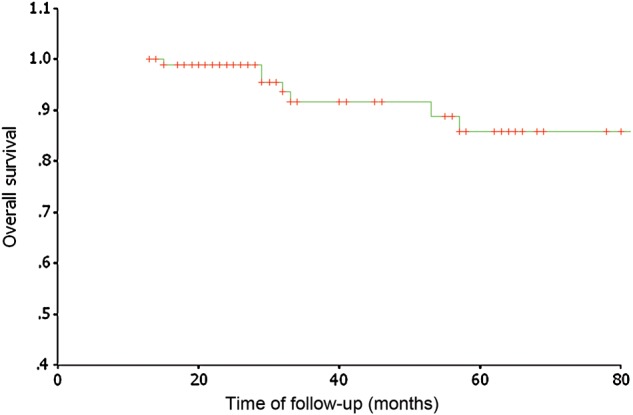
Overall survival for the 97 patients treated with SMART.

At the time of the analysis, treatment failure was seen in 18 patients. Eight (8/18) of these patients experienced local recurrence, two (2/18) had cervical lymph node recurrence, nine (9/18) had local-regional recurrence, and ten (10/18) developed distant metastasis. The details of treatment failure of the patients are shown in Table 3. All local recurrences were in-field failures within the nasopharynx gross target volume. For the patients with local recurrence, we gave a second course of radiation with IMRT and achieved satisfactory results; five (5/8) patients are still alive without tumor recurrence. For the patients with cervical lymph node recurrence, we did selective neck dissection; the two patients are alive and still under observation. For patients suffering from distant metastasis, we gave them radiation therapy, stereotactic body radiation therapy (SBRT) and/or chemotherapy; four of these patients are still alive and six patients died of metastastic disease. The 5-year LCR was 93.3%. For locally advanced disease (T3 and T4), the 3-year and 5-year LCRs were 91.7% and 69.9%, respectively (shown in Fig 1). At the endpoint of observation 12 patients had died. Of these, six patients died because of distant metastasis and two patients died of other chronic disease, one from a second malignancy, and the other from cardiac disease one month after treatment. Four patients died of nasopharyngeal hemorrhage. Of these, the nasopharyngeal hemorrhage was caused by local recurrence in two patients, and the other two patients had ulcers induced by a second course of radiotherapy given to the nasopharynx. The DMFS and OS rates were 90.3% and 91.6% at 3 years, and 87.9% and 85.7% at 5 years, respectively (shown in Figs 2 and 3). For 38 patients with Stage I–II disease, the 3-year and 5-year LCRs were 91.4% and 91.4%, respectively; the DMFS and OS rates were 100% and 100% at 3 years, and 94.1% and 93.8% at 5 years.

**Table 3.** Patients with treatment failure

Patient (T&N stage)	Local (interval^a^)	Cervical lymph node (interval)	Local-regional (interval)	Distant metastasis (interval)	Living status
1 (T1N0)	+^b^(14 mo^c^)		+(14 mo)		Dead
2 (T3N2)	+(20 mo)	+(20 mo)	+(20 mo)	+(20 mo)	Dead
3 (T3N2)	+(49 mo)		+(49 mo)		Dead
4 (T1N2)				+(10 mo)	Dead
5 (T3N0)	+(36 mo)		+(36 mo)		Living
6 (T2N1)	+(18 mo)		+(18 mo)		Living
7 (T2N1)				+(40 mo)	Dead
8 (T2N3)				+(3 mo)	Living
9 (T4N1)				+(9 mo)	Dead
10 (T2N3)		+(7 mo)	+(7 mo)		Living
11 (T2N0)	+(21 mo)		+(21 mo)		Living
12 (T4N3)				+(8 mo)	Dead
13 (T4N3)				+(16 mo)	Living
14 (T3N2)				+(20 mo)	Living
15 (T4N2)	+(9 mo)		+(9 mo)		Living
16 (T4N2)	+(16 mo)		+(16 mo)		Living
17 (T4N2)				+(15 mo)	Living
18 (T1N3)				+(8 mo)	Dead

^a^Indicates time from the date of the end of treatment to the date of failure.

^b^Indicates existence. ^c^mo = month.

**Fig. 4.**
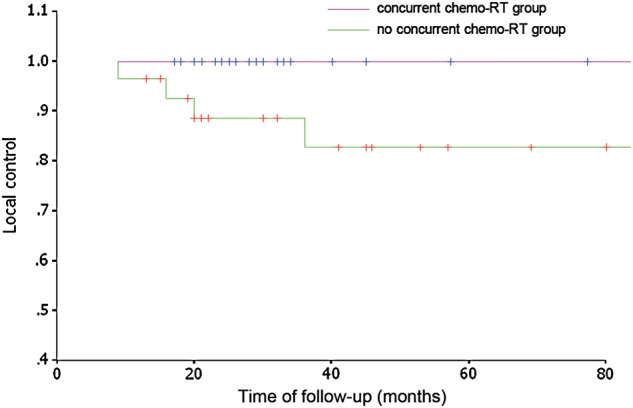
Local control for the two groups of patients treated with SMART.

**Fig. 5.**
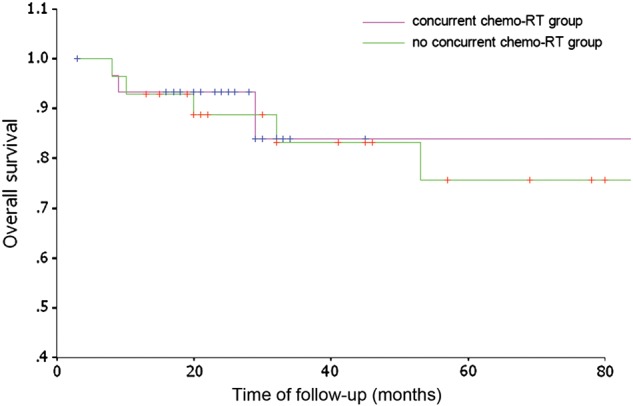
Overall survival for the two groups of patients treated with SMART.

In addition, 59 patients with Stage III and IV disease were analyzed. Of these, 31 patients received concurrent chemoradiotherapy, whereas 28 patients received radiotherapy without concurrent chemoradiotherapy. Of these 28 patients, six patients received induction chemotherapy and 22 patients received adjuvant chemotherapy, the mean number of cycles given being 1.2 and 2.8, respectively. Of the 59 patients with Stage III and IV disease, the 3-year and 5-year LCRs were 94.6% and 81.9%; the DMFS and OS rates were 82.1% and 83.9% at 3 years, and 82.1% and 76.9% at 5 years, respectively (shown in Figs 4 and 5). The details of treatment results of the patients are shown in Table 4.

**Table 4.** Five-year survival in the 59 patients with Stage III/IVa/IVb disease

	With concurrent chemo-RT	No concurrent chemo-RT	*P*-value
LC	100%	82.6%	0.38
DMFS	82.0%	82.0%	0.95
OS	84.0%	75.7%	0.91

The most commonly observed severe (i.e. Grade 3 or 4) acute toxicities included Grade 3 mucositis, skin desquamation, and leucopenia, which occurred in 78 (80.4%), 8 (8.2%), and 45 (46.4%) of the 97 patients in the study, respectively. No patient had a stomach tube inserted or a gastrostomy before or during treatment for nutritional support. Grade 3 acute skin toxicity developed in 10% of the patients, but no Grade 4 skin toxicity was observed. One patient presented Grade 4 leucopenia. The total incidence of Grade 3 or 4 acute toxicities in patients receiving IMRT plus concurrent chemotherapy was 86.0%, significantly higher than those receiving IMRT alone (59.8%) (*P* < 0.005).

Late toxicities were assessed in 40 patients with > 3 years follow-up. The results are depicted in Table 5. The most common late toxicities were hearing loss, trismus and xerostomia. No patient had Grade 3–4 late toxicity. Xerostomia was assessed in all patients during follow-up. Grade 1 xerostomia had developed in 47.5% of patients and Grade 2 xerostomia in 5% of patients at a median follow-up of > 36 months. The severity of xerostomia diminished over time after treatment. The number of patients with Grade 2–3 xerostomia decreased gradually, whereas the number of patients with Grade 0–1 xerostomia increased in the follow-up period. Three patients had Grade 2 trismus, but no Grade 3–4 trismus was noted. No patient suffered neuropathy, brainstem injury, mandible necrosis or temporal lobar injury. Although three patients had significant memory deterioration, the MRIs did not show any change in temporal lobar injury.

**Table 5.** The frequency of late toxicities for 40 patients with >3 years of follow-up

Type	Grade 0, *n* (%)	Grade 1, *n* (%)	Grade 2, *n* (%)	Grade 3, *n* (%)	Grade 4, *n* (%)
Hearing loss	22	11	7	0	0
Xerostomia	19	19	2	0	0
Trismus	26	11	3	0	0
Chronic dysphagia	40	0	0	0	0
Neuropathy	40	0	0	0	0
Temporal lobe necrosis	40	0	0	0	0
Brainstem injury	40	0	0	0	0
Mandible necrosis	40	0	0	0	0

## DISCUSSION

IMRT has been used since the mid-1990s to treat different head and neck cancers, including NPC. Due to the ability to spare normal structures and improve dosage to the tumor volume, IMRT has become the treatment of choice for NPC. IMRT techniques can generally provide more conformal plans, especially for irregular or concave-shaped target volumes in the vicinity of critical structures. Non-randomized comparative studies on IMRT vs either conventional RT (2D-RT) or 3-dimensional conformal RT ( 3D-CRT) plans for the treatment of NPC have demonstrated the superiority of IMRT in achieving better tumor coverage and sparing of organs at risk (OARs) such as the central nervous system, parotid glands and mandible [14–17]. These features make IMRT the treatment of choice in clinical situations where there is a dose–response relationship between tumor control, overall treatment time [18–20] and radiation dose delivered to the tumor [21–25]. To yield a higher biologically equivalent dose, dose escalation or altered fraction schemes could be given. We developed an IMRT with SMART boost techniques regimen of 2.33 Gy per fraction for a total of 69.9 Gy for the treatment of NPC. With this strategy, the whole course of treatment can be done by one plan, and different dose levels can be delivered to different targets simultaneously during the same treatment period. Dose escalation to the tumor can also be achieved through the delivery of higher fractional doses and shortening of overall treatment time, which could result in improved local tumor control. Based on a linear–quadratic equation with an α/β ratio of 10 for tumor response, escalation of the mean biologic equivalent dose (BED) for the gross target volume reached 86.2 Gy, which resulted in an 8.6% increase in the BED to the boost volume (the 2 Gy per fraction equivalent of 71.8 Gy). The fraction dose to the nasopharynx gross target volume was increased to 2.33 Gy, and the overall treatment time was shortened by 1 week. With a median follow-up of 42 months, a preferable clinical efficacy with less late toxicity was confirmed. The 5-year local control rate was 87.6%. The most common late toxicities were Grades 1–2. No Grade 4 late toxicities were noted.

Over the last decade, there have been several reports published concerning the use of IMRT with SMART boost techniques for the treatment of patients with NPC [9, 26–33]. It seems that the short-term results reported in those series were all quite good. However, the fraction dose ranged from 2.09–2.5 Gy, and the total physical dose ranged from 68–79.5 Gy. After we transformed the physical dose into BED, the BED ranged from 83.4–97.5 Gy. We found that the BED of the nasopharynx gross target volume in our series was similar to the BEDs in Bakst *et al.*'s series [33], which were 86.2 Gy and 86.6 Gy, respectively, whereas the BEDs reported by other authors ranged from only 83.4–84.8 Gy [27–28, 30, 32]. By contrast with their result, our result seems to be quite good. Some series [26, 29], whose BEDs were much higher than ours, have greater LCRs than ours, while Xiao *et al.*'s [9] result was no better than ours, although the BED in their study was obviously higher than ours. Compared with studies that only enrolled patients with advanced TNM classification [9, 29, 33], our outcomes still seem to be better. Many T4 patients remain free of disease at last follow-up, suggesting that they might have benefited from the hypofractionated approach. The results are detailed in Table 6. Furthermore, most of the studies integrate other techniques to boost the dose after IMRT, such as brachytherapy [26, 31], SRS [29], 3DRT, and IMRT [27, 28]. Considering all the above factors, the local control achieved in our study is quite satisfactory, with 3- and 5-year LCRs of 93.3% and 87.6%, respectively. This implies that IMRT with the SMART boost technique, using our dose scheme to enhance the BED of the nasopharynx gross target volume, may be a better choice to improve the local control of disease for patients with NPC.

**Table 6.** Summary of the reported use of the SMART boost technique for NPC

Author, year	*n*	Stage III/IV (*n*) (AJCC)	Boost after IMRT	Fractionation scheme for GTV of nasopharynx				LCR% (duration of follow-up)
				Fraction	Total dose, Gy	Fraction dose, Gy	BED, Gy	
Lee *et al.*, 2002	67	29	Yes	33	74.6	2.26	91.4	97 (4 years)
Kam *et al.*, 2004	63	32	Yes	33	69	2.09	83.4	92 (3 years)
Wu *et al.*, 2006	75	42	Yes	28	68	2.43	84.6	97.3 (2 years)
Kwong *et al.*, 2006	50	50	Yes	35	79.5	2.27	97.5	95.7 (2 years)
Koom *et al.*, 2008	24	22	No	27	67.5	2.5	84.4	93 (3 years)
Lin *et al.*, 2009	323	198	Yes	31	72.2	2.33	89.1	95 (3 years)
RTOG0225, 2009	68	23	No	33	70	2.12	84.8	92.6 (2 years)
Bakst *et al.*, 2011	25	25	No	30	70.2	2.34	86.6	91 (3 years)
Xiao *et al.*, 2011	81	81	No	30	73.8	2.46	92	94.9 (3 years)
Current study	97	59	No	30	69.9	2.33	86.2	93.3 (3 years) 94.6% (3 years) for Stage III/IV cases

IMRT = intensity-modulated radiotherapy, GTV = gross tumor volume, Gy = gray, BED = biologically effective dose, LCR = local control rate, AJCC = American Joint Committee on Cancer.

Chemotherapy also has an important role in the treatment of NPC alongside RT. Intergroup 0099 established the role for chemoradiotherapy in the treatment of NPC [12]. Multiple randomized Phase III trials that followed have shown an advantage for concurrent chemo-RT in locally advanced NPC, although the chemotherapy generally consisted of cisplatin-based chemotherapy in most of the studies [5, 34–37]. Chitapanarux [38] used carboplatin instead of cisplatin for concurrent chemoradiotherapy to treat 206 locoregionally advanced NPC patients, and although the compliance of patients in the carboplatin group was better, no significant difference was noticed in treatment efficacy between these two arms. Kwong [39] reported a trial which used uracil and tegafur (600 mg daily) as concurrent with adjuvant chemotherapy of CDDP/5FU + VBM for three cycles; they found a significant improvement in the DMFS (*P* = 0.026), with a similar trend in both OS (87% vs 77%) and progression free survival (69% vs 58%). But attention should also be given to compliance and toxicity accompanying concurrent and adjuvant chemotherapy, because within the intergroup 0099 trial only 55% of the patients completed adjuvant chemotherapy [12]. The chemotherapy regimen we used contained 135 mg/m^2^ paclitaxel on Day 1 and 25 mg/m^2^ cisplatin on Days 1–3 instead of cisplatin for concurrent chemotherapy, and the same regimen was also applied to induction chemotherapy and adjuvant chemotherapy, which is widely used for head and neck cancers. Toxicity was acceptable in both groups. Although a trend of improvement in LCRs (100% vs 82.6%) was seen, there was no statistically significant difference between the concurrent chemo-RT group and the no chemo-RT group for LCR, DMFS or OS. Adequate chemotherapy has been shown by meta-analysis [40] to decrease local/distant failure and to improve the survival of patients with locoregionally advanced NPC. In our regimen, the paclitaxel dose was reduced to 135 mg/m^2^ on Day 1 and the cisplatin dose was reduced to 25 mg/m^2^ on Days 1–3 to reduce toxicity. Therefore, we speculate that the limited chemotherapy used in this trial is quite likely to be the reason for the less than satisfactory LCR, DMFS and OS rates. A higher-dose chemotherapy regimen for a larger population of patients will be included in a further study. In our study 31 patients received concurrent chemotherapy, the 5-year OS was 84.0%, and distant metastasis was the major cause of death in patients after treatment. Although concurrent chemotherapy is generally accepted to be the standard of care for locoregionally advanced NPC, the best chemotherapy regimen remains debatable.

In our study, we found that the accelerated regimen did result in increased acute toxicities of the skin and mucous membranes, but with anti-inflammatory therapy and nutrition support, no patient required significant treatment breaks and all patients finished treatment. We also observed a few significant late toxicities. Grades 1 and 2 toxicities were observed in 40 patients over 3 years of follow-up, but no patient had Grade 3–4 toxicity. The lower incidence of deafness, trismus, temporal lobe necrosis, neuropathy, dysphagia and xerostomia in our current cohort was especially dramatic compared with the long-term toxicities in previous retrospective and prospective investigations [41, 42]. As was reported in other studies [26, 27], xerostomia appeared to decrease with time after treatment. The incidence of trismus was quite low. Several reports [9, 33] have indicated the occurrence of temporal lobe necrosis. It is worthwhile to note that no temporal lobe necrosis was detected in our trial, although 12% of patients developed this kind of injury in Bakst *et al.*'s trial [33] (in which patients with NPC received almost the same technique of radiotherapy as our patients). Temporal lobe necrosis may be the result of extensive invasion of the skull base and even intracranial tissue, which led to increased doses to the temporal lobes. We found that the ratio of patients with T4 disease in the two trials were similar (21.6% vs 20.0%), however, only three (3.1%) of the patients suffered from intracranial extension in our group. Also, Bakst *et al.* found the median time till detection of temporal lobe necrosis after radiation was 24 months (range, 19–48 months). For the three patients with intracranial extension in our group, the follow-up times were 14 months, 25 months and 37 months, respectively, which was relatively shorter, I think. Maybe those above were the difference between the two trials. However, the specified dose constraints to the temporal lobes and a modified PTV to limit the dose to the temporal lobes were still rather important. So in our view, the regimen of 2.33 Gy for a total of 69.9 Gy with the use of IMRT with SMART boost techniques still seems to be safe with respect to temporal lobe necrosis. All these results suggest that potential functional gains from SMART boost IMRT with our fractionation scheme restricted the dose delivered to the normal tissue around the tumor and resulted in decreased rates of severe late toxicities, thus improving the quality of life.

## CONCLUSIONS

Long-term outcomes for NPC patients who underwent IMRT with the SMART boost technique in our study demonstrate excellent LCR, DMFS and OS with acceptable and tolerable treatment-related toxicities. However, no improvement was noted in LCR, DMFS or OS using concurrent chemoradiotherapy that contained paclitaxel and cisplatin as our chemotherapy regimen, and distant metastasis was still the major cause of treatment failure in our study. Although further research is required, there is a trend in the clinical data that suggests an enhanced therapeutic ratio and a possible survival advantage for this modality of radiation therapy. A larger population of patients and a longer follow-up period are needed to evaluate ultimate tumor control and late toxicity. New strategies identifying the best dosage for an optimal chemotherapy regimen to enhance the therapeutic effect of chemoradiotherapy need to be developed in further studies.
